# Supplementary figures and images for: Heat and cold stress increases the risk of paroxysmal supraventricular tachycardia
Source: PLoS One. 2024 Jan 2;19(1):e0296412. doi: 10.1371/journal.pone.0296412 (PMC10760728; doi:10.1371/journal.pone.0296412)

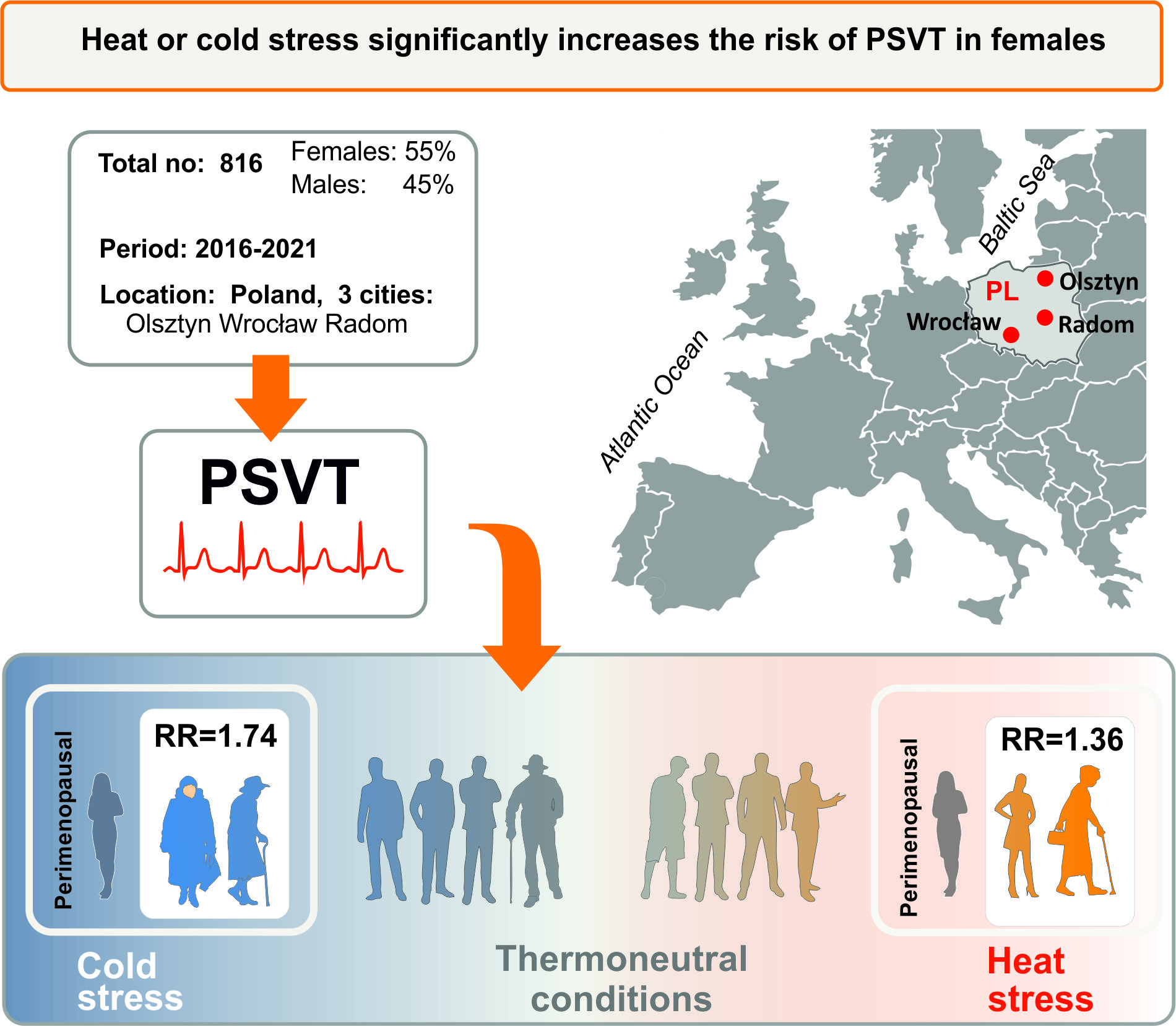

Supplement: S1 Graphical abstract — (TIF) [file pone.0296412.s003.tif]
