# Supplementary material for: Heat and cold stress increases the risk of paroxysmal supraventricular tachycardia
Source: PLoS One. 2024 Jan 2;19(1):e0296412. doi: 10.1371/journal.pone.0296412 (PMC10760728; doi:10.1371/journal.pone.0296412)
Supplement: Attachment — Submitted filename: Response to Reviewers.docx [file pone.0296412.s004.docx]

**Response to Reviewers' and Editors’ comments**

We want to thank the Reviewers for their insightful reviews of our manuscript. Please, find below the Reviewers’ comments written in bold and our responses inserted after each of their comments. The responses refer to the line numbering from the original (first) version. Next, our response to editorial issues is prepared in this same way.

**Reviewer 1**

**Although the role of climate in diseases is undoubtedly important, I consider that in such a large study many other variables need to be assessed that are impossible to do due to the nature of the study.**

*AD. We treat this remark as a comment rather than a suggestion to correct the text. We share the reviewer's opinion, this type of research always has a certain risk of secondary dependencies. We write about it in the limitations section. We agree with the reviewer that climatic factors are among the increasingly important determinants of many diseases. Therefore, we would like to emphasize the applicability of UTCI when climate-related diseases are of concern.*

**Reviewer 2**

*AD. Reviewer 2 does not raise any critical comments. We want to thank him for his positive attitude to our article.*

**Minor comments: for table 1 and 3, maybe put down years below the age?**

*AD. Corrected according to suggestion and consequently Fig. 3 as well.*

**Reviewer 3**

**The gaps from previous research, then justify your research to explain the innovations made. then add the purpose of making this research and explain what contribution is obtained to the public, add justification and contribution to the last paragraph.**

*AD. The last paragraph of the discussion section has been supplemented with the relevant explanations as suggested by the Reviewer.*

*“Our study extends the knowledge about the relationship between climate and humans. The results demonstrate that the use of an appropriate tool, in this case the comprehensive biometeorological index (UTCI), can help to find relationships with PVST that have not been described previously. From a medical point of view, we have proven the hypothesis that an increased incidence of PSVT occurs more often after extreme thermal episodes.”*

**Editors’ comments**

**1. When submitting your revision, we need you to address these additional requirements. Please ensure that your manuscript meets PLOS ONE's style requirements, including those for file naming.**

*AD. The text has been formatted according to the Journal requirements.*

**2. Did you know that depositing data in a repository is associated with up to a 25% citation advantage…**

*AD. Our database is available as S1 Table in supplementary material*

**3. We note that Figure 1 in your submission contain map images which may be copyrighted. All PLOS content is published under the Creative Commons Attribution License (CC BY 4.0), which means that the manuscript, images, and Supporting Information files will be freely available online, and any third party is permitted to access, download, copy, distribute, and use these materials in any way, even commercially, with proper attribution. For these reasons, we cannot publish previously copyrighted maps or satellite images created using proprietary data, such as Google software (Google Maps, Street View, and Earth).**

*AD. The graphics (including the borders of Europe) in Figure 1 have been adapted and modified from the authors' private, unpublished material. Figure 1 was created by the authors in CorelDraw based on the data collected during the study. It has never been published before.*

**4. Please include captions for your Supporting Information files at the end of your manuscript, and update any in-text citations to match accordingly.**

*AD. Done.*

We remain at your disposal.

On behalf of all authors,

Jerzy Romaszko
